# Experience with Mammitis*Read before the N. Y. State Academy of Veterinary Science and Comparative Pathology.

**Published:** 1885-10

**Authors:** Chas. A. Meyer


### ART. XXVI.—EXPERIENCE WITH MAMMITIS.*

BY CHAS. A. MEYER, D.V.S.

The subject of this paper will be understood by most veterinary practitioners, and I believe one which is very difficult of treating satisfactorily, especially for the owners of dairy cattle, and is known as mastitis, mammitis, caked bag, garget, and sundry vague terms. It is an acute inflammation of the mammary gland, or "udder."

The disease is due to the most diverse causes—lactation is the principal predisposing cause—and appears immediately before or soon after parturition; sometimes it is the result of external injuries, such as blows, or injuries caused while milking, as well as many others too numerous to mention. One other common cause is overstocking, which consists in the preparation of the cow for show, or market, by allowing her to go unmilked, until the udder becomes enormously distended with milk, and which is done to give the gland a fine appearance; at times it again arises through not thoroughly milking dry, the milk remaining in the gland, and acting as an irritant.

My experience has been mainly in cow-sheds which were in a filthy condition, and am satisfied that mammitis is due to putrid infection, and believe that the majority of cases are of septic origin. Much could be said on this subject, but experience is better than theory, as a specific treatment will give good results; my views have been, and are now, that in the majority of cases mammitis is purely a form of erysipelas of the quarters of the mammæ.

The disease rarely attacks the whole gland, but is usually confined to one or two quarters; the affected parts are hot, hard, swollen, tender, and red; the skin is tense and shining; the quarter is enlarged, hard and sensitive to manipulation; some parts feel lumpy from the presence of coagula; by stripping or mulsion of the teat a rose-tinted fluid is obtained, with strings of clotted milk, and usually mixed with blood.

---

* Read before the N. Y. State Academy of Veterinary Science and Comparative Pathology.

The constitutional symptoms depend upon the severity of the attack, the disease often being ushered in with rigors; the bowels are usually costive, and at other times unusually loose, the muzzle more or less dry, appetite slightly impaired, pulse quickened, together with general fever.

The prognosis, as given in text-books, is generally unfavorable, and for the first few years of my experience, the treating of the disease was most certainly discouraging. I resorted to all authors, where mention was made in their work, and followed their treatment, and invariably the quarters of the cow affected were lost for the season. I finally resorted to homœopathy, and treated with their specific phytolacca decandra, or poke root, but with no better results; ointments of various kinds were applied, until finally I gave up all hope of ever finding a remedy, and refused to treat any more affected cows—those to whom I was called advised the owners to sell for slaughter.

In the spring of 1882, while pleuro-pneumonia was raging quite severely, I was again called into cattle practice, and came across odd cases of mammitis. I read more deeply, and thought still stronger that there must be some relief for these animals, and finally came to the conclusion that there might be a possibility that the disease might be erysipelas, *per se.;* as Ferri Sesqui Chloridum is a specific in human medicine, why should not a mineral tonic in cattle be a specific as well? I set about treating some cases, and gave directions to give one powder, dissolved in water, *ter in die;* to strip the cow every hour or two, to hand rub the gland with soft soap, and to make a poultice of bran and soft soap, to be applied hot, and to keep changing every hour for six to eight hours; after this to bathe the gland with hot soap water three times a day, and during intervals to apply the soap to the affected parts. The question then arose, where to get the soft soap. I said, make it; Pearline powder will make the best I know of, and is easy of application. About thirty-six to forty-eight hours after the treatment had been used orders came to my office: need not call; cows are all right.

My fee had not been paid, and I naturally thought: well, I will see who has the cases now. I called, was treated rather cool; when the first party said: well, the cows are all right.

Well, was my reply, I called to satisfy myself; don't have any fear, this visit costs you nothing. I came to collect my fee, as it is not mnch, and to satisfy myself that the cows are well. I was shown into the shed, and, sure enough, my patients were nearly well, giving their full quantity of milk, and looking healthy. Since then my success has been the same, and under this treatment have yet to see a case where failure is the reply, with this treatment.

The only other remedy used internally was and is the following:

℞

Cupri. Sulph., ℥jj

Nucis Vomicæ, ℥jss

M. ft. Pulv. No. viii.

Liq. Am. in water t. i. d.

There are many forms of the disease spoken of, and it is very difficult to differentiate between them. I have used the same treatment before parturition, and when the calf was born it did the stripping, and my results were always very satisfactory.

